# Microscopy and transcriptomic datasets for investigating the drought-stress response and recovery in young and early senescent-old leaves from *Brassica napus*

**DOI:** 10.1016/j.dib.2024.111130

**Published:** 2024-11-14

**Authors:** Pierre-Nicolas Boulc'h, Vanessa Clouet, Gevorg Ghukasyan, Marie-Françoise Niogret, Laurent Leport, Maja Musse

**Affiliations:** a UMR Institut de Génétique, Environnement et Protection des Plantes (IGEPP), INRAE, Institut Agro Rennes-Angers, Université Rennes, Le Rheu, France; b UR Optimisation des Procédés en Agro-alimentaire, Agriculture et Environnement (OPAALE), INRAE, Rennes, France; c Plateforme Biosit Histo pathology High precision (H2P2), Université Rennes, Rennes, France

**Keywords:** RNA sequencing, Differential gene expression, Leaf cross section, Mesophyll tissue, Winter oilseed rape, Water stress, Abiotic stress

## Abstract

The present dataset combines transcriptomic and microscopic analyses to investigate the responses of winter oilseed rape (WOSR, Brassica napus L., cultivar Aviso) to soil drought, with a focus on differences between young and early-senescent old leaves. For microscopy, 36 scans of 1 to 5 leaf cross-sections were acquired from paraffin-embedded leaf disc samples using a scanner with a 40x lens (Pannoramic Confocal, 3DHistech), capturing a large field of view (8-mm-long observed leaf tissue). The raw scanned cross-sections and analyzed images are available under doi.org/10.57745/RK5PM3 in the Recherche Data Gouvrepository. These high-quality scans enable the differentiation of mesophyll cells and tissues. Software analysis yielded a dataset with 54 selected cross-sectional areas, 291 delimited surfaces of palisade, spongy, and vessel tissues, and 11,136 individually delimited cells from the palisade and spongy layers. For transcriptomics, an Illumina Novaseq sequencer was used to generate 390 Gb of mRNA paired-end reads. The raw reads were filtered, mapped, and assigned to genes from the Brassica napus reference genome *Darmor-bzh* v10, which were subsequently used to identify differentially expressed genes (DEGs) and to perform gene ontology enrichment analysis. The raw reads are accessible under accession PRJNA939927 at the NCBI Sequence Read Archive (SRA). This high-quality dataset provides insights into the molecular mechanisms underlying oilseed rapeʼs response to soil drought and may aid in the development of drought-tolerant cultivars. A total of 17,975 DEGs were identified between well-watered and severe drought conditions across the contrasted leaf developmental stages.

Specifications Table

**Table:** 

Subject	Biological Sciences
Specific subject area	**Plant Science:** Plant Physiology
Type of data	Table, Graph, Figure
Data collection	The data presented here originate from samples of *Brassica napus* leaves taken from three distinct developmental stages: very young (Y1), young (Y2), and early-senescent old (O). The leaves were collected from plants that had been grown under well-watered conditions (WW) or subjected to progressively intensifying soil drought, which resulted in medium (MD), high (HD), and severe (SD) drought conditions, followed by a two-day rewatering phase (RW). Microscopy images (36 scans, each containing one to five cross-sections) were obtained from paraffin-embedded leaf samples from the selected leaves using a scanner equipped with a 40x lens (Pannoramic Confocal, 3DHistech), providing a large field of view (8 mm of observed leaf tissue). The construction and sequencing of the RNA libraries were conducted by Genewiz/Azenta, NGS Lab, Germany. The libraries were prepared using the NEBNext Ultra II RNA Library Prep Kit for Illumina, following the manufacturer's instructions (NEB, Ipswich, MA, USA). The sequencing was performed on the Illumina NovaSeq™ 6000 platform, yielding an average of approximately 60 million 2 × 150 bp paired-end reads per sample.
Data source location	Institution: Institute for Genetics, Environment and Plant Protection (IGEPP) City: Le Rheu Country: France Latitude and longitude: 48°6′32.643″N, 1°47′34.436″W
Data accessibility	Repository name: Recherche Data Gouv & NCBI Sequence Read Archive (SRA) Data identification number: 10.57745/RK5PM3 (microscopy) & 10.57745/7HQSM3 (transcriptomic, SRA PRJNA939927) Direct URL to data: … https://entrepot.recherche.data.gouv.fr/dataset.xhtml?persistentId=doi:10.57745/RK5PM3 (microscopy) https://entrepot.recherche.data.gouv.fr/dataset.xhtml?persistentId=doi:10.57745/7HQSM3 (transcriptomic) https://www.ncbi.nlm.nih.gov/bioproject/PRJNA939927 (transcriptomic)
Related research article	*[**1**] Boulc'h, P.-N., Clouet, V., Niogret, M.-F., Avice, J.-C., Musse, M. & Leport,* L. *(2024) Leaf drought adaptive response in winter oilseed rape is altered at the onset of senescence: a study combining NMR relaxometry, multi-omics and microscopy. Physiologia Plantarum, 176(4), e14454. Available from:*10.1111/ppl.14454

## 1 Value of the Data

• The present study provides both microscopy images and a transcriptomic dataset of leaves at three developmental stages from winter oilseed rape (WOSR, *Brassica napus* L.*, cv. Aviso*) plants that were well-watered or subjected to a progressive soil drought followed by a recovery phase.
• These data are valuable to the scientific community working on plant physiology, especially on leaf development, drought adaptation, and post-drought recovery.
• The microscopy dataset may be particularly useful for investigating changes in the mesophyll structure of WOSR leaves under different stress conditions and developmental stages. Further analysis of the microscopic images may provide new information about structural changes in vascular tissues and epidermis during water stress responses on soil drought, which may support breeding programmes for developing drought-tolerant WOSR cultivars.
• The transcriptomic dataset may be particularly useful for characterising changes in the transcriptional expression of WOSR genes at different leaf developmental stages and/or at different water stress intensities and may serve as a resource for identifying drought-responsive candidate genes. Further analysis of the transcriptomic dataset may also lead to new information regarding the gene sub-functionalization processes related to leaf development or water stress responses, potentially providing insights for breeding programmes focused on the development of drought-tolerance WOSR cultivars.

## 2 Background

Ongoing climate change is disrupting crop yield stability, particularly by reducing soil moisture and thus water availability to crops [2]. Therefore, describing the adaptive responses of plants to these stresses is a current and future challenge for the identification of tolerance traits to be integrated in varietal selection programmes. The aim of this dataset was to characterise both the structural and transcriptomic responses of WOSR leaves at different developmental stages under progressive soil drought.

The microscopy dataset includes images from three developmental stages of leaves from plants that were well-watered or subjected to low, medium, high and severe soil drought, followed by a recovery phase. This dataset is valuable for studying mesophyll structural changes and general mechanisms of leaf development, drought response and post-drought recovery. In parallel, the transcriptomic dataset includes gene expression data from leaves at the same developmental stages and water stress conditions. This dataset provides insights into the molecular responses associated with leaf development and stress adaptation.

While the associated research paper [1] mainly focuses on the physiological responses, i.e. leaf water status and mesophyll structural changes occurring during the drought response, the current dataset provides a broader perspective for investigating both structural changes and differentially expressed genes associated with leaf development, drought and post-drought recovery general mechanisms.

## 3 Data Description

**Fig. 1:**
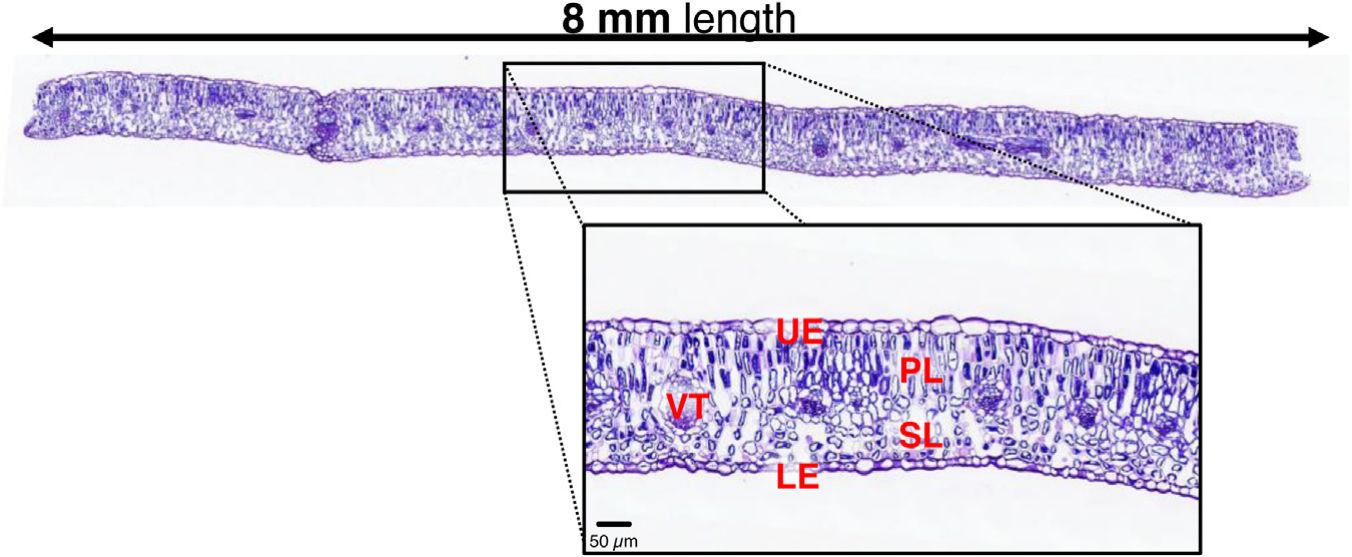
Example of visualisation of total scanned area from leaf cross-sections. Visualisation was carried out with the CaseViewer software (Version used: 2.4.0.119028, 3DHISTECH). UE, upper epidermis; LE, lower epidermis; PL, palisade layer; SL, spongy layer; VT, vascular tissue.

**Fig. 2:**
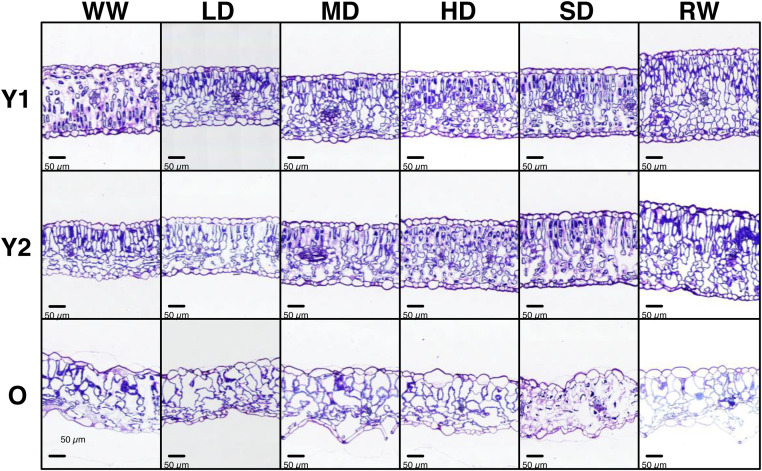
Selection of representative areas from scanned leaf cross-sections at three developmental stages (Y1, very young leaves; Y2, young leaves; O, early senescent-old leaves) from winter oilseed rape (WOSR, *Brassica napus* L., cv. Aviso) plants grown under different soil conditions (WW, well-watered; LD, low drought; MD, medium drought; HD, high drought; SD, severe drought; RW, re-watered).

**Fig. 3:**
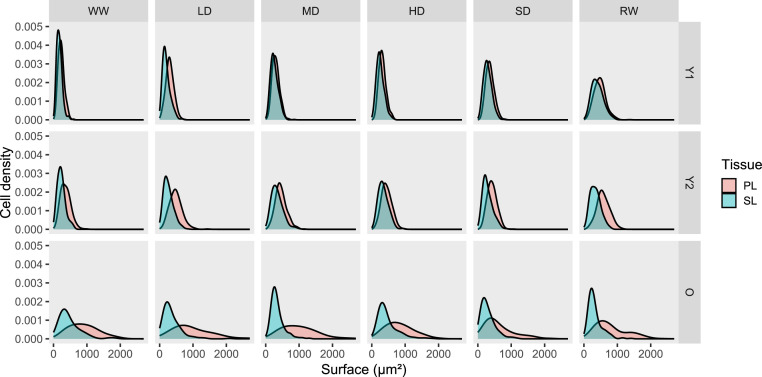
Cell surface density from palisade (PL) and spongy (SL) layer measured with the CaseViewer software (Version used: 2.4.0.119028, 3DHISTECH) from scanned leaf cross-sections at three developmental stages (Y1, very young leaves; Y2, young leaves; O, early senescent-old leaves) from winter oilseed rape (WOSR, *Brassica napus* L., cv. Aviso) plants grown under different soil conditions (WW, well-watered; LD, low drought; MD, medium drought; HD, high drought; SD, severe drought; RW, re-watered).

The provided image scans include 1 to 5 cross-sections of 8 mm-long leaves, as shown in Fig. 1. The high quality of the scans enables to differentiate individual cells in the upper (UE) and lower (LE) epidermis, palisade (PL) and spongy (SL) parenchyma, and vascular tissue (VT). Under well-hydrated conditions (WW), differences in mesophyll structure were observed between young (Y1 and Y2) and early senescent-old (O) leaves (Fig. 2). During drought, the mesophyll structure of young leaves remained stable, while that of early senescent-old leaves deteriorated. After soil rewatering, the mesophyll thickness of young leaves (Y1 and Y2) strongly increased, while no changes were observed for early senescent-old leaves. Using software analysis, we selected 54 leaf cross-sections areas and delimited 291 surfaces of palisade, spongy, and vascular tissues, as well as 11,136 distinguishable cells from both palisade and spongy layers. In young leaves (Y1 and Y2), the PL and SL cells showed a similar surface distribution (Fig. 3). In early senescent-old leaves, the surface distribution of PL cells was wider, and the majority of PL cells were larger than SL cells. Leaf development induced substantial changes in cell size distribution, while soil drought induced slight changes.

**Fig. 4:**
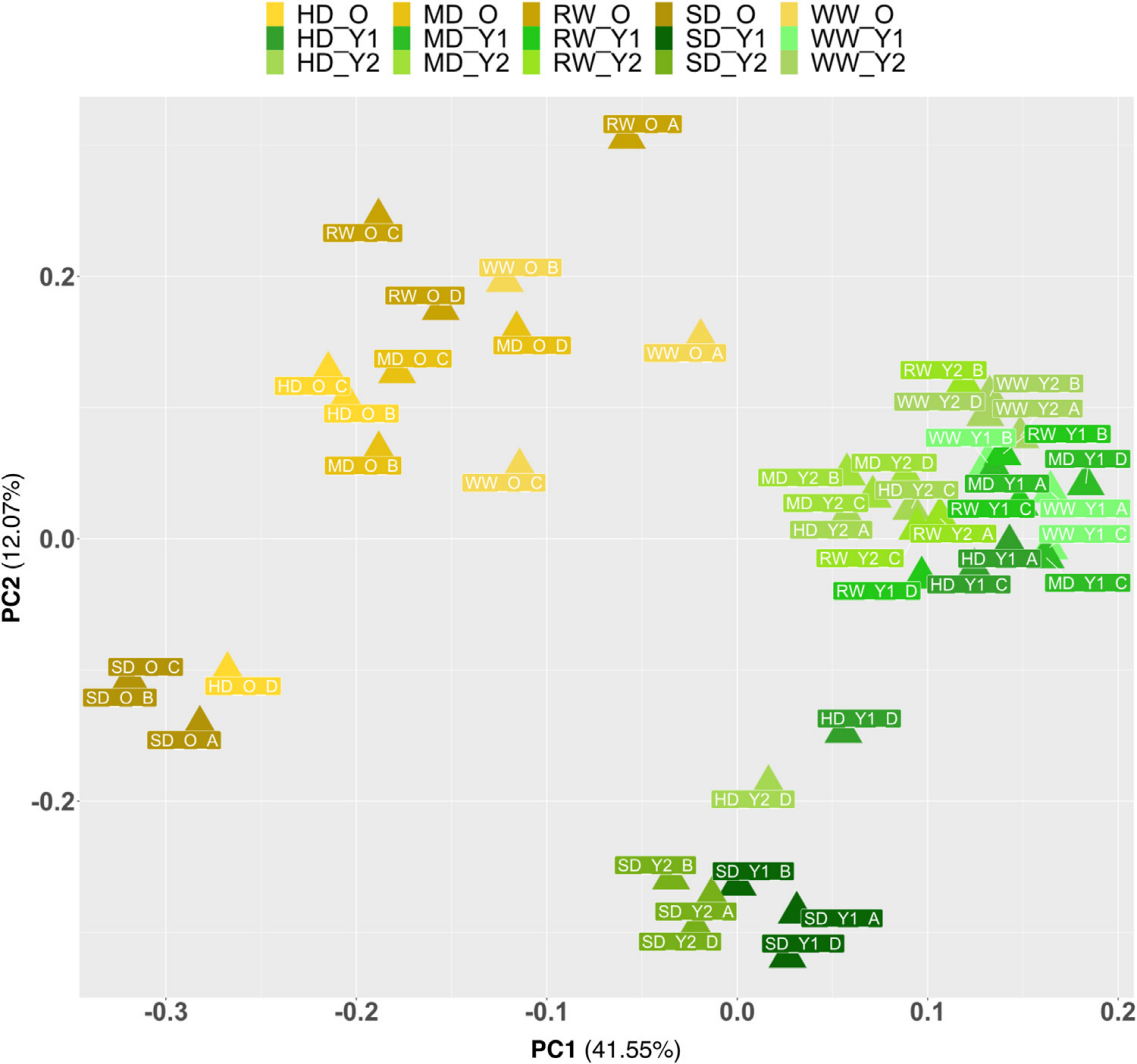
Principal Component Analysis (PCA) score plots generated using transcriptomic dataset obtained from very young (Y1), young (Y2), and early senescent-old (O) leaves of winter oilseed rape (WOSR, *Brassica napus* L., cultivar Aviso) plants grown under different soil conditions (WW, well-watered; MD, medium drought; HD, high drought; SD, severe drought; RW, re-watered). The biological replicates are denoted by letters (A–D) in the name of the samples.

**Fig. 5:**
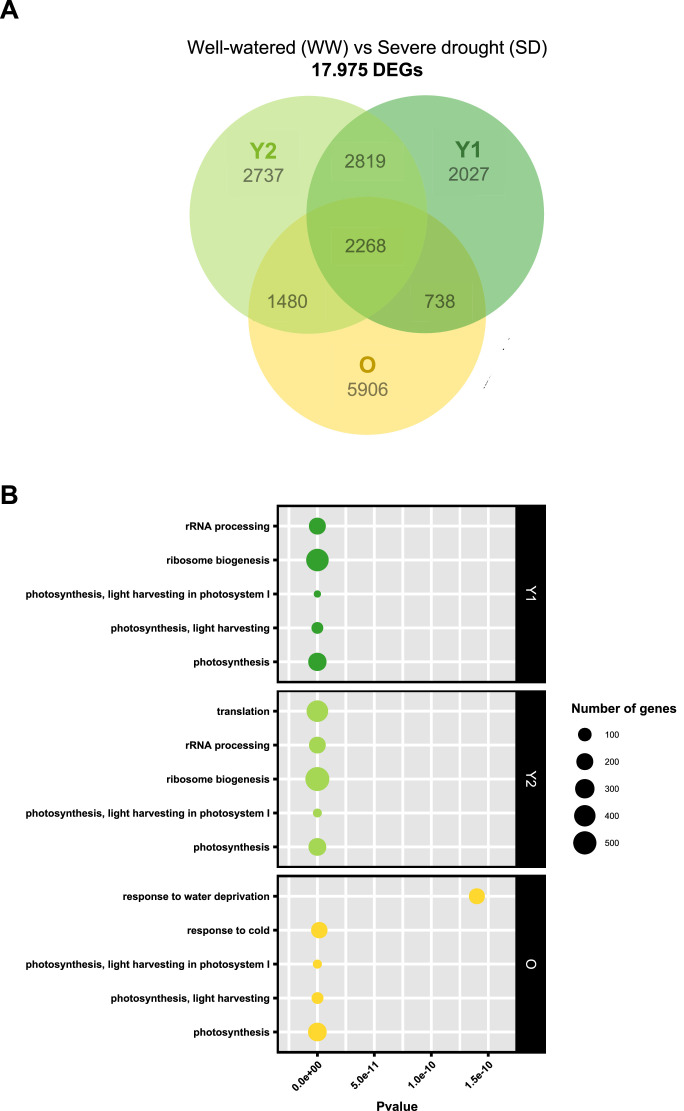
Venn diagram distribution (A) of 17,975 differentially expressed genes (DEGs) identified in the three leaf developmental stages (Y1, very young leaves; Y2, young leaves; O, early senescent-old leaves) between winter oilseed rape (WOSR, *Brassica napus* L., cultivar Aviso) plants grown under well-watered (WW) and severe drought (SD) soil conditions. Dot plot (B) of the 5 most represented biological functions among the 17,975 DEGs identified for the three selected leaf developmental stages between WOSR plants grown under WW and SD soil conditions. The enriched Gene Ontology (GO) terms were identified using the reference genome *Darmor-bzh* v10 of *Brassica napus* [3]. The size of the dots is related to the number of genes involved in each function.

The transcriptomic dataset is of high quality (Table 1), with an average sequencing depth of 59.5 ± 10.7 million clean reads (2 × 150 bp each) and a mean quality score (QC) of 35.2 ± 0.1 per sample. Among the clean reads pairs, 91.6 ± 2.2 % were mapped and 66.8 ± 5.6 % assigned to a unique annotated gene from the *Brassica napus* reference genome *Darmor-bzh* v10 [3]. Principal component analysis shows a good separation between expression profiles of early senescent-old and young leaves (Fig. 4). Good separation is also observed between soil conditions, since SD plants are strongly differentiated from other soil conditions. A total of 17,975 DEGs were identified in the three selected developmental stages between WW and SD plants (Fig. 5A). Among these, 10,392 DEGs were identified in early senescent-old leaves, slightly more than for Y2 (9304) and Y1 (7852). 5906 DEGs were early senescent-old leaves-specific, while 4486 DEGs were shared by early senescent-old and young leaves. On the other hand, Y1 and Y2 shared 5087 DEGs, when 2737 and 2027 DEGs were specific of Y2 and Y1 respectively. The annotation of DEGs highlighted biological functions that were the most impacted by severe stress (Fig. 5B), and especially photosynthesis for all leaf developmental stages. Translation and ribosome biogenesis mechanisms seems primarily affected in young leaves, while early senescent-old leaves mainly exhibit responses known to be linked to water and temperature stresses.

**Table 1:** Summary of RNA-seq samples, including the number of million clean reads, the mean quality score (QC) and the percentage of mapped read pairs assigned to unique annotated genes in the *Brassica napus* reference genome *Darmor-bzh* v10 [3]. Sample IDs are given according to soil condition (WW; well-watered soil; MD, medium soil drought; HD, high soil drought; SD, severe soil drought; RW, re-watered soil), leaf development stage (Y1, very young leaf; Y2, young leaf; O, early senescent-old leaf) and replicate letter (A–D).

Sample ID	Number of million clean reads	Mean QC	% mapped read pairs	% assigned to annotated genes
WW-O-A	74.7	35.1	92.2	67.5
WW-O-B	67.1	35.3	93.3	72.2
WW-O-C	45.9	35.1	92.0	66.9
WW-Y1-A	70.8	35.1	90.7	63.1
WW-Y1-B	45.7	35.1	93.4	69.3
WW-Y1-C	70.3	35.2	91.2	60.3
WW-Y2-A	57.7	35.1	91.2	62.0
WW-Y2-B	45.3	35.0	92.6	66.4
WW-Y2-D	65.4	35.1	90.4	59.7
MD-O-B	68.5	35.1	92.6	70.3
MD-O-C	66.1	35.2	92.6	69.0
MD-O-D	71.7	35.2	93.8	73.8
MD-Y1-A	53.5	35.2	84.7	61.8
MD-Y1-C	48.4	35.1	92.3	65.6
MD-Y1-D	71.3	35.3	92.4	64.6
MD-Y2-B	65.2	35.2	92.5	66.8
MD-Y2-C	72.4	35.3	92.7	67.1
MD-Y2-D	62.6	35.1	91.2	67.1
HD-O-B	47.3	35.2	92.7	70.4
HD-O-C	60.2	35.3	94.2	77.1
HD-O-D	51.4	35.2	94.0	74.1
HD-Y1-A	51.2	35.2	91.3	65.5
HD-Y1-C	51.5	35.2	92.0	66.8
HD-Y1-D	49.2	35.2	92.1	67.6
HD-Y2-A	56.7	35.2	91.9	67.3
HD-Y2-C	65.5	35.1	90.8	63.4
HD-Y2-D	47.5	35.2	91.9	67.2
SD-O-A	78.2	34.9	88.3	63.1
SD-O-B	62.9	35.2	87.0	66.0
SD-O-C	49.2	35.2	92.9	71.4
SD-Y1-A	47.2	35.2	89.8	57.2
SD-Y1-B	79.1	35.1	90.5	63.4
SD-Y1-D	59.9	35.0	86.7	54.9
SD-Y2-A	46.1	35.2	90.7	56.0
SD-Y2-B	83.9	35.2	88.5	63.5
SD-Y2-D	45.2	35.1	89.5	62.1
RW-O-A	45.6	35.2	94.9	75.7
RW-O-C	49.9	35.2	94.0	76.8
RW-O-D	53.5	35.3	94.7	77.0
RW-Y1-B	61.0	35.2	92.8	70.2
RW-Y1-C	58.5	35.3	93.1	68.8
RW-Y1-D	63.7	35.1	87.4	58.5
RW-Y2-A	64.9	35.1	91.0	63.3
RW-Y2-B	69.9	35.3	94.2	76.4
RW-Y2-C	57.0	35.2	93.2	70.3
Mean	59.5	35.2	91.6	66.8
SD	10.7	0.1	2.2	5.6

## 4 Experimental Design, Materials and Methods

### 4.1 Plant material, growth conditions and experimental design

Approximately 300 seeds of winter oilseed rape (WOSR, *Brassica napus* L.*,* cultivar Aviso) were individually weighed. The 100 most uniform seeds were selected and placed on moistened absorbent paper in a Petri dish. After 3 days, around 80 seedlings were chosen based on visual criteria, including root development, cotyledon emergence, and overall uniformity. These seedlings were then individually transplanted into 9L plastic pots (Airpot®) filled with a mixture of sandy loam (40 % v/v), peat moss (60 % v/v), added clay (40 kg.m^-3^), and NPK (0.7 kg.m^-3^ PG-MIX 14-16-18) with a soil solution at pH 5.8 ± 0.2.

All plants were grown and equally distributed into 4 randomized blocks within a semi-controlled environment in a greenhouse (Latitude and longitude: 48°6′32.643″N, 1°47′34.436″W) equipped with an air-cooling system, with a temperature / relative humidity / photoperiod of 25 °C / 75 % / 14h days and 18 °C / 90 % / 10 h nights. Natural light was supplemented to ensure a minimum of 200 µmol.m^-2^.s^-1^ of photosynthetically active radiation (PAR) at canopy height. From 0 to 55 days after sowing (DAS), all non-vernalized plants were watered at a rate of 450 mL / day of fertilized water (0.3 % Liquoplant Bleu, 2.5 % N, 5% P, 2.5 % K) distributed throughout the day using 3 drippers per pot connected to an automatic valve system. Soil humidity was monitored by capacitive soil moisture probes (YARA ZIM Plant Technology GmbH) and was maintained at 30–40 %.

From 55 to 68 DAS, half of the 44 most uniform plants at the rosette stage with 10 true leaves were selected to undergo a period of water withholding to induce soil drying down to 1 % soil humidity. At 68 DAS, the plants were rewatered with 1L of fertilized water before restarting automatic watering until 70 DAS. During the water-withholding period, plants produced 3 new leaves (13 leaf ranks in total at 68 DAS). One additional leaf was produced during rewatering phase (14 leaf ranks in total at 70 DAS). Leaf samples were taken at 55, 58, 59, 64, 68 and 70 DAS from plants under well-watered (WW, 30 % soil humidity), low drought (LD, 12 % soil humidity), medium drought (MD, 8 % soil humidity), high drought (HD, 3 % soil humidity), severe drought (SD, 1 % soil humidity) and rewatered (RW, 20 % soil humidity) soil condition, respectively.

### 4.2 Leaf stage selection and sampling

Three biological replicates of very young (Y1), young (Y2), and early senescent-old (O) leaves were selected from all WW, LD, MD, HD, SD, and RW plants. Their respective developmental stages were characterized on the basis of their nuclear magnetic resonance (NMR) signal as previously described [4,5], therefore preventing from developmental stage effects between different sampling dates and taking into account the new leaves production during experiment [1]. At the time of harvest, 24 leaf discs (8 mm²) were dedicated for *in vivo* NMR analysis, one leaf disc (8 mm²) was sampled for microscopy and a ¼ segment of intact limb was frozen in liquid nitrogen and stored at -80 °C for transcriptomic.

### 4.3 Light microscopy at large field of view

A fixative solution (phosphate buffer 0.2 mol.L-1 at pH 7.4 with 4 % (v/v) formaldehyde) was infiltrated into the leaf discs using ten cycles of depressurization with a vacuum pump (5 min of low pressure in each cycle). Samples were returned to the same fixative solution at 4 °C for storage. The day of preparation, leaf discs were inserted between two sponges to keep them flat within cassettes printed using PrintMate (Thermo Fischer Scientific). The tissue samples were then processed for paraffin embedding using the Excelsior ES machine (Thermo Fischer Scientific) for 9 h and 44 min under vacuum and agitation, with successive increasing alcohol baths (ref. 83813.360, VWR), xylene (ref. 28973.363, VWR), and paraffin at 62 °C (ref. 10048502, Qpath). Tissue were then embedded in paraffin with the use of Histostar (Thermo Fischer Scientific). Paraffin-embedded tissues sections of 3 µm were cut using a microtome (HistoCore Multicut, Leica Biosystems), and mounted onto glass slides. The slides were placed in an oven at 56 °C for at least 1 h to improve adhesion of the sections to the slides. For staining, the slides were deparaffinized and the sections were manually stained with toluidine blue solution (ref. 115930, Merck) at room temperature for 1 min. The toluidine blue solution was prepared in advance by dissolving 10 g Toluidine Blue in 750 mL distilled water with 5 g lithium carbonate. Once dissolved, 640 mL glycerol and 50 mL 95 % ethanol were added. The solution was then filtered through a 0.45 µm filter before use. The slides were washed under running water until the dye stopped diffusing into the water, dehydrated, and mounted with PERTEX glue (ref. 00811-EX, Histolab) between a glass slide and a cover slip. Cross section images of paraffin-embedded leaf disc samples were then acquired using a scanner with a x40 lens (Pannoramic Confocal, 3DHistech), allowing a very large field of view (8mm-long observed leaf tissue).

### 4.4 Image analysis

Images were analysed using CaseViewer software (Version used: 2.4.0.119028, 3DHISTECH). First, a framework of 865 µm in length and 460 µm in width was defined for all images. Within these frames, the boundary of the palisade (PL) and spongy (SL) layers and the vascular tissues (VT) were manually delimited. Then each individual cell belonging to PL and SL was manually delimited and the mean cell area was computed for each tissue. The surface of intercellular spaces was estimated by subtracting the sum of the areas of the PL and SL cells and VT from the surface of the PL and SL layers.

### 4.5 RNA isolation and sequencing

The frozen leaf samples were ground, and RNA extractions were conducted using NucleoZOL with NucleoSpin® RNA Set for NucleoZOL in accordance with the manufacturer's instructions (MachereyNagel GmbH & Co., Düren, Germany). To remove any genomic DNA contamination, a DNA digest step in solution was performed using rDNase Set (Macherey-Nagel). Subsequently, the RNA preparations were purified using the NucleoSpin® RNA Clean-up kit (Macherey-Nagel). The quality of RNA was assessed using a NanoDrop® One^C^ Microvolume UV–vis Spectrophotometer (NanoDrop Technologies, Wilmington, DE, USA) and a 2100 Bioanalyzer® (Agilent Technologies, Santa Clara, CA, USA), yielding mean values for OD260/280 = 2.16, OD260/230 = 2.2, RIN = 6.04, 28S/18S = 0.8. A control PCR was performed on RNA extracts to ensure the absence of genomic DNA. All RNA samples were sent to the Genewiz-Azenta NGS Lab in Leipzig, Germany. RNA sequencing libraries were prepared using the NEBNext Ultra II RNA Library Prep Kit for Illumina, following the manufacturer's instructions (NEB, Ipswich, MA, USA). The average insert length for library preparation was ∼200nt. Libraries of the 45 samples were sequenced using the Illumina NovaSeq sequencing platform of the Azenta lab, producing an average of 60 M of 2 × 150 bp reads per sample and a mean quality score (QC) of 35.2.

### 4.6 RNAseq data analyses

Processing of the transcriptomic raw data was carried out using the adapted nf-core RNA-seq pipeline version 3.8.1 ([6]; https://github.com/nf-core/rnaseq). Briefly, the pipeline was based on Nextflow v22.04.0 [7] and the data processed using the default options with the following steps: (1) quality control with FastQC v0.11.9 [8] prior and after trimming with TrimGalore! v0.6.7 [9], (2) read mapping onto the reference genome *Darmor-bzh* v10 [3] with STAR v2.7.10a [10] and (3) transcript quantification with featureCounts v1.6.0 [11] according to the *Darmor-bzh* v10 gene annotation. Additional quality tools were used: RseQC v3.0.1 [12], Preseq v3.1.1 [13], dupRadar v1.18.0 [14], DESeq2 v1.28.0 [15]. The pipeline output files were plotted and summarised with multiQC tools v1.8 [6]. For gene expression analyses, the AskoR pipeline v1.0.0 ([16]; https://github.com/askomics/askoR) was used with the following steps: (1) data filtering with a threshold of 2 cpm (count per million), (2) data normalization, (3) visualization of correlations, and (4) differential expression analysis with a relative log expression (RLE) normalization method [15] and a quasi-likelihood function for the GLM model (Generalized Linear Model of the negative binomial family). A threshold of 0.05 was set for the Benjamini Hochberg p-value adjustment [17] and 1 for the log2 Fold-Change value. All statistical analyses were achieved with RStudio software.

## Limitations

None.

## Ethics Statement

The authors have read and follow the ethical requirements for publication in Data in Brief and confirm that the current work does not involve human subjects, animal experiments, or any data collected from social media platforms*.*

## Credit Author Statement

**Pierre-Nicolas Boulc'h:** Methodology, Investigation, Visualization, Writing - Original Draft, **Vanessa Clouet:** Data curation, Formal analysis, Investigation, Visualization, Writing - Original Draft, **Gevorg Ghukasyan:** Investigation, **Marie-Françoise Niogret:** Supervision, Conceptualization, Writing - Review & Editing, **Maja Musse:** Supervision, Conceptualization, Writing - Review & Editing, **Laurent Leport:** Supervision, Conceptualization, Writing - Review & Editing.

## Data Availability

Research Data GouvTranscriptomic dataset for investigating the drought-stress response and recovery in young and early senescent-old leaves from Brassica napus (Original data). NCBI Sequence Read ArchiveTranscriptomic dataset for investigating the drought-stress response and recovery in young and early senescent-old leaves from Brassica napus (Original data). Research Data GouvLight microscopy dataset for investigating the drought-stress response and recovery in young and early senescent-old leaves from Brassica napus (Original data).
